# Supplementary material for: Iterative improvement in the automatic modular design of robot swarms
Source: PeerJ Comput Sci. 2020 Dec 7;6:e322. doi: 10.7717/peerj-cs.322 (PMC7924708; doi:10.7717/peerj-cs.322)
Supplement: Supplemental Information 3 [file peerj-cs-06-322-s003.zip › argos3/doc/api/standalone/a00615_source.html]

ARGoS: plugins/simulator/physics\_engines/dynamics2d/dynamics2d\_stretchable\_object\_model.cpp Source File


- Main Page
- Related Pages
- Namespaces
- Classes
- Files

- File List
- File Members

# plugins/simulator/physics\_engines/dynamics2d/dynamics2d\_stretchable\_object\_model.cpp

Go to the documentation of this file.

```
00001 #include "dynamics2d_stretchable_object_model.h"
00002 #include "dynamics2d_gripping.h"
00003 
00004 namespace argos {
00005 
00006    /****************************************/
00007    /****************************************/
00008 
00009    CDynamics2DStretchableObjectModel::CDynamics2DStretchableObjectModel(CDynamics2DEngine& c_engine,
00010                                                                         CComposableEntity& c_entity) :
00011       CDynamics2DSingleBodyObjectModel(c_engine, c_entity),
00012       m_fMass(0.0f),
00013       m_pcGrippable(NULL),
00014       m_ptLinearFriction(NULL),
00015       m_ptAngularFriction(NULL) {}
00016 
00017    /****************************************/
00018    /****************************************/
00019 
00020    CDynamics2DStretchableObjectModel::~CDynamics2DStretchableObjectModel() {
00021       if(m_pcGrippable != NULL) delete m_pcGrippable;
00022       if(m_ptLinearFriction != NULL) {
00023          cpSpaceRemoveConstraint(GetDynamics2DEngine().GetPhysicsSpace(), m_ptLinearFriction);
00024          cpConstraintFree(m_ptLinearFriction);
00025       }
00026       if(m_ptAngularFriction != NULL) {
00027          cpSpaceRemoveConstraint(GetDynamics2DEngine().GetPhysicsSpace(), m_ptAngularFriction);
00028          cpConstraintFree(m_ptAngularFriction);
00029       }
00030    }
00031    
00032    /****************************************/
00033    /****************************************/
00034 
00035    void CDynamics2DStretchableObjectModel::MoveTo(const CVector3& c_position,
00036                                                   const CQuaternion& c_orientation) {
00037       if(m_pcGrippable != NULL) {
00038          /* Release all attached entities */
00039          m_pcGrippable->ReleaseAll();
00040       }
00041       CDynamics2DSingleBodyObjectModel::MoveTo(c_position, c_orientation);
00042    }
00043    
00044    /****************************************/
00045    /****************************************/
00046 
00047    void CDynamics2DStretchableObjectModel::Reset() {
00048       if(m_pcGrippable != NULL) {
00049          /* Release all attached entities */
00050          m_pcGrippable->ReleaseAll();
00051       }
00052       CDynamics2DSingleBodyObjectModel::Reset();
00053    }
00054    
00055    /****************************************/
00056    /****************************************/
00057 
00058    void CDynamics2DStretchableObjectModel::SetLinearFriction(Real f_max_bias,
00059                                                              Real f_max_force) {
00060          m_ptLinearFriction =
00061             cpSpaceAddConstraint(GetDynamics2DEngine().GetPhysicsSpace(),
00062                                  cpPivotJointNew2(GetDynamics2DEngine().GetGroundBody(),
00063                                                   GetBody(),
00064                                                   cpvzero,
00065                                                   cpvzero));
00066          m_ptLinearFriction->maxBias = f_max_bias;
00067          m_ptLinearFriction->maxForce = f_max_force; // emulate linear friction
00068    }
00069    
00070    /****************************************/
00071    /****************************************/
00072 
00073    void CDynamics2DStretchableObjectModel::SetAngularFriction(Real f_max_bias,
00074                                                               Real f_max_force) {
00075          m_ptAngularFriction =
00076             cpSpaceAddConstraint(GetDynamics2DEngine().GetPhysicsSpace(),
00077                                  cpGearJointNew(GetDynamics2DEngine().GetGroundBody(),
00078                                                 GetBody(),
00079                                                 0.0f,
00080                                                 1.0f));
00081          m_ptAngularFriction->maxBias = f_max_bias;
00082          m_ptAngularFriction->maxForce = f_max_force; // emulate angular friction
00083    }
00084 
00085    /****************************************/
00086    /****************************************/
00087 
00088 }
```

---

Generated on 10 Jul 2018 for ARGoS by 
 1.6.1
